# Bone morphogenetic protein 9, and its genetic variants contribute to susceptibility of idiopathic pulmonary arterial hypertension

**DOI:** 10.18632/aging.102726

**Published:** 2020-02-07

**Authors:** Kefang Guo, Liying Xu, Lin Jin, Huilin Wang, Yun Ren, Yan Hu, Jing Yu, Jing Cang

**Affiliations:** 1 Department of Anesthesiology, Zhongshan Hospital, Fudan University, Shanghai 200032, China

**Keywords:** pulmonary arterial hypertension, polymorphism, BMP9, biomarker

## Abstract

Considering the predominant role of rare variants of the Bone morphogenetic protein 9 (BMP9) gene in the occurrence of idiopathic pulmonary arterial hypertension (IPAH), here we conducted a case-control study, together with functional validation, to explore the relationships between variants of the BMP9 gene and development of IPAH. We found minor alleles of rs3740297 (OR: 0.72, 95% CI: 0.59-0.87, P=7.77×10-5) and rs7923671 (OR: 0.76, 95% CI: 0.62-0.93, P=0.009) were significantly associated with decreased risk of IPAH. Minor alleles of rs3740297 and rs7923671 were significantly associated with increased plasma level of BMP9 in both IPAH cases and controls (P<0.001). An allele of rs7923671 showed higher relative luciferase activity compared to that containing G allele (P<0.001). Mechanism exploration found that pulmonary artery smooth muscle cells (PASMC) cell line transfected with rs3740297 C allele construct, miR-149 mimic, and antagomir miR-149 showed more sensitive change of the relative luciferase activity and BMP9 expression. This means minor allele T of rs3740297 could significantly decrease susceptibility of IPAH in Chinese population, possibly by increasing BMP9 expression through losing a miR-149 binding site. Our study provides evidence for genetic associations between two specific variants in the BMP9 gene and plasma level of BMP9, occurrence of IPAH.

## INTRODUCTION

Pulmonary arterial hypertension (PAH), a severe vasculopathy characterized by progressive narrowing and obliteration of the pulmonary arterioles, resulted in heart failure and premature death, and had raised the public concerns [1–3]. PAH was still associated with high rate of severe cardiovascular events and dismal mortality, although the treatment of PAH has advanced substantially over the past 20 years [4, 5]. It was estimated that the incidence of PAH ranges from 2.0 to 7.6 cases per million adults per year, and its prevalence varies from 11 to 26 cases per million adults worldwide [6]. Although growing evidence revealed multiple genetic and environmental mechanisms contributed to PAH development, including the incapacitation or mutation of the bone morphogenetic protein receptor type-2 (*BMPR2*) gene, epigenetic abnormality, or the sex hormone imbalance, the exact pathogenesis still remains unclear [6]. These factors could only explain a fraction of PAH cases, suggesting additional PAH genes awaiting exploration [6, 7].

Previous studies had supported a pivotal contribution of Bone morphogenetic protein 9 (*BMP9*, also known as growth differentiation factor 2) signaling axis to PAH [8–11]. Homozygous nonsense mutations in *BMP9* had been reported in a child with severe PAH [11]. Recently, Wang et al. [9] conducted an exome-wide gene-based burden analysis and reported that the rare coding mutations in *BMP9* gene occurred in 6.7% of idiopathic PAH (IPAH) cases, ranking this gene second to the *BMPR2* gene. Later study in Caucasians also provide independent validation of a critical role for *BMPR2* in PAH [12]. Besides, *BMP9* was also identified as a sensitive and specific biomarker of porto-pulmonary hypertension (POPH) [10]. Considering the predominant role of rare variants of the *BMP9* gene in the occurrence of PAH, none studies have explored the contributions of the common variants of the *BMP9* gene to the pathogenesis susceptibility of IPAH. Thereby, here we conducted a case-control study, together with functional validation, to explore the relationships, if any, between common variants of the *BMP9* gene and development of IPAH.

## RESULTS

### Characteristics of study population

The characteristics of 836 patients with IPAH and 900 control subjects included in this study were shown in Table 1. There were no significant differences in the distribution of age, gender, smoking and drinking status, as well as BMI between the IPAH cases and controls (P > 0.05 for all). These results indicate the comparability of this case-control study design.

**Table 1:** Distributions of selected variables in IPAH cases and healthy controls.

	**Cases (n=836)**	**Controls (n=900)**	**P value**
Age			
<40	384 (45.9%)	451 (50.1%)	0.082
≥40	452 (54.1%)	449 (49.9%)	
Gender			
female	559 (66.9%)	593 (65.9%)	0.667
male	277 (33.1%)	307 (34.1%)	
Smoking status			
Yes	162 (19.4%)	160 (17.8%)	0.391
No	674 (80.6%)	740 (82.2%)	
Drinking status			
Yes	214 (25.6%)	226 (25.1%)	0.816
No	622 (74.4%)	674 (74.9%)	
BMI (Kg/m2)	25.7±6.3	25.3±6.1	0.179

BMI: body mass index.

### The relationships of the *BMP9* SNPs in IPAH risk

The genotypic distribution of the five selected tagSNPs of the *BMP9* gene among the IPAH cases and controls was shown in Table 2. Genotype frequencies of five tagSNPs in controls were all in agreement with HWE (P > 0.05). Among the five SNPs, we found minor alleles of rs3740297 (OR: 0.72, 95% CI: 0.59-0.87, P=7.77×10^-5^) and rs7923671 (OR: 0.76, 95% CI: 0.62-0.93, P=0.009) were significantly associated with decreased risk of IPAH. For rs3740297, the adjusted OR for the carriers with the CT genotype was 0.76 (95% CI: 0.64-0.91, P=0.003) and for those with the TT genotype was 0.51 (95% CI: 0.32-0.82, P=0.005), compared with the CC genotype. While for rs7923671, the adjusted OR for the carriers with the AG genotype was 0.78 (95% CI: 0.61-1.00, P=0.051) and for those with the AA genotype was 0.46 (95% CI: 0.23-0.95, P=0.034), compared with the GG genotype.

**Table 2:** Genetic variants of the *BMP9* gene and susceptibility of IPAH.

**SNP**	**PAH cases**	**Controls**	Adjusted OR (95% CI)*	**P value**
rs3740297				
CC	601	578	1.00 (reference)	
CT	213	279	0.76 (0.64-0.91)	0.003
TT	22	43	0.51 (0.32-0.82)	0.005
T vs C			0.72 (0.59-0.87)	7.77×10^-5^
rs7923671				
GG	612	615	1.00 (reference)	
AG	213	262	0.78 (0.61-1.00)	0.051
AA	11	23	0.46 (0.23-0.95)	0.034
A vs G			0.76 (0.62-0.93)	0.009
rs73299055				
TT	742	799	1.00 (reference)	
TC	89	98	1.02 (0.82-1.27)	0.880
CC	5	3	1.87 (0.44-7.87)	0.396
C vs T			1.07 (0.54-2.1)	0.850
rs3781226				
CC	604	656	1.00 (reference)	
CT	219	229	1.08 (0.71-1.64)	0.718
TT	13	15	0.98 (0.76-1.26)	0.868
T vs C			1.06 (0.64-1.77)	0.817
rs4922508				
CC	715	764	1.00 (reference)	
CT	117	131	0.99 (0.95-1.03)	0.721
TT	4	5	0.89 (0.35-2.28)	0.807
T vs C			0.99 (0.94-1.04)	0.678

* Adjusted for age, gender, smoking and drinking status, and BMI

P value in bold means statistically significant.

### Effect of *BMP9* rs3740297 and rs7923671 on plasma level of *BMP9*

**Figure 1:**
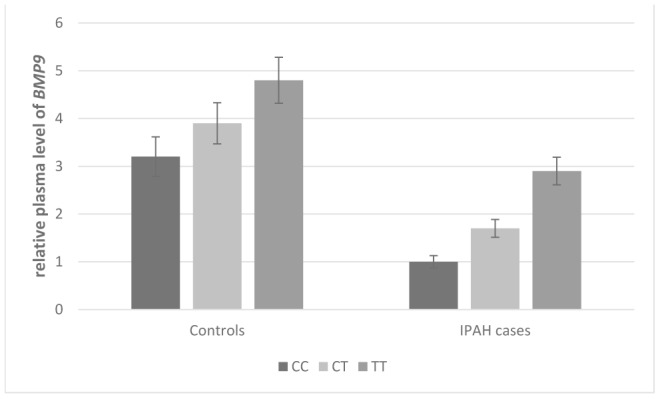
**Effect of *BMP9* rs3740297 on plasma level of *BMP9*.** The plasma levels of *BMP9* were relative to those with major homozygotes in IPAH cases.

**Figure 2:**
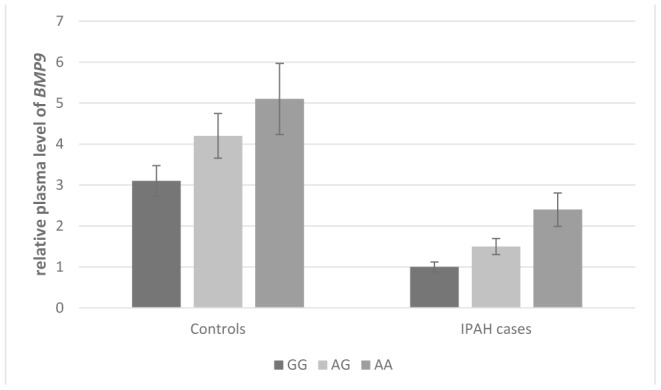
**Effect of *BMP9* rs7923671 on plasma level of *BMP9*.** The plasma levels of *BMP9* were relative to those with major homozygotes in IPAH cases.

As shown in Figures 1 and 2, the effect of *BMP9* rs3740297 and rs7923671 on plasma level of *BMP9* was presented. The plasma level of *BMP9* in IPAH cases was significantly lower than that in controls (P<0.001). Next, we examined whether any of the two polymorphisms were correlated with plasma level of *BMP9*. We found minor alleles of rs3740297 and rs7923671 were significantly associated with increased plasma level of *BMP9* in both IPAH cases and controls (P<0.001).

### Effect of *BMP9* rs3740297 and rs7923671 on transcriptional activity

**Figure 3:**
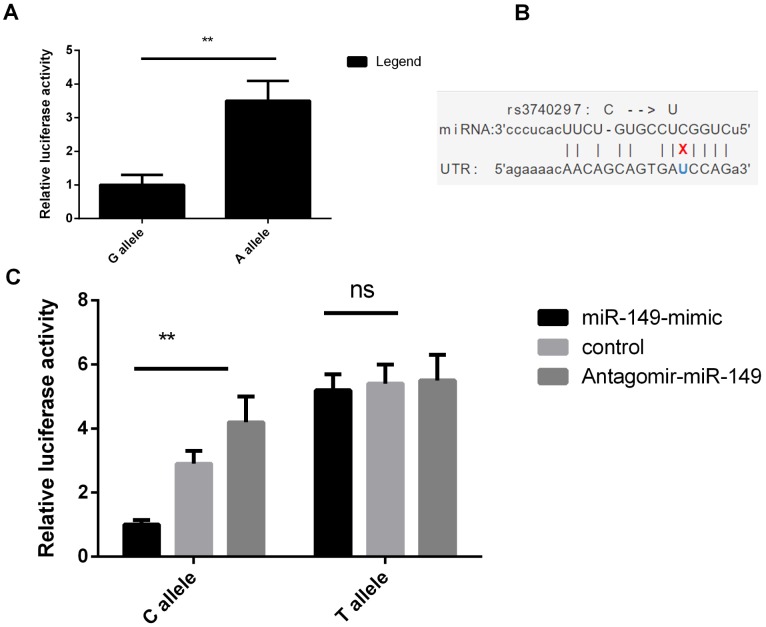
***BMP9* rs7923671 and rs3740297 affect luciferase activities in PASMC cells.** (**A**) relative luciferase activities of rs7923671 were measured in PASMC cells. (**B**) SNP-miRNA duplex for rs3740297 and miR-149. (**C**) relative luciferase activities of rs3740297 were measured in PASMC cells transfected with C allele, or T allele. Cells in different groups were treated with blank control, miR-149 mimic or antagomir-miR-149. Three replicates for each group and the experiment were repeated three times.

**Figure 4:**
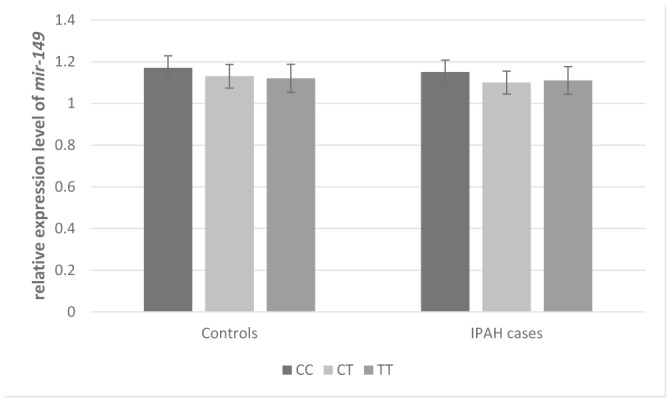
**Effect of *BMP9* rs3740297 on plasma level of *MIR-149*.** The plasma levels of *BMP9* were relative to those with major homozygotes in IPAH cases.

**Figure 5:**
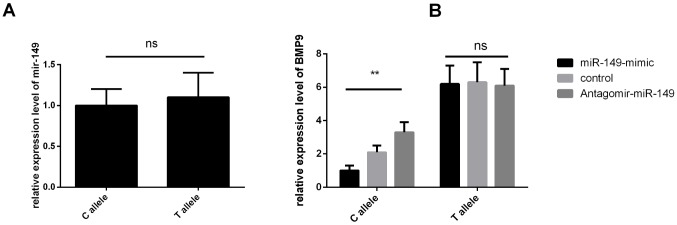
***BMP9* rs3740297 affect l expression level of *MIR-149* and the *BMP9* gene in PASMC cells.** (**A**) relative expression level of *MIR-149* were measured in PASMC cells. (**B**) relative expression level of the BMP9 gene were measured in PASMC cells transfected with C allele, or T allele. Cells in different groups were treated with blank control, miR-149 mimic or antagomir-miR-149. Three replicates for each group and the experiment were repeated three times.

HaploRegv4.1 and miRNASNP V2 were used to annotate the functional elements for *BMP9* rs3740297 and rs7923671. We found that rs7923671 was located at a locus with multiple transcription factor (Myc, Pax-2, Pou5f1, P300) binding site, and rs3740297 was located at the 3’ UTR region of the *BMP9* gene and its C>T transition could cause a binding site loss of miR-149 (Figure 3B), which suggest their potential allele-specific regulatory effect. To verify our hypothesis, we first measured the expression level of miR-149 in plasma of and PASMC cells, respectively. As shown in Figure 4, the expression level of miR-149 was not influenced by the genotypes of SNP rs3740297, as well as the disease status of IPAH (P >0.05). Meanwhile, the PASMC cells transfected with vectors containing the C allele of rs3740297 showed identical expression level of miR-149, compared to those containing the T allele (Figure 5A, P >0.05).

Then, we conducted the dual-luciferase reporter assay to validate the bioinformatics analysis findings. As shown in Figure 3, the PASMC cell lines transfected with vectors containing the A allele of rs7923671 showed higher relative luciferase activity compared to that containing the G allele (P<0.001, Figure 3A), which confirmed its allele-specific regulatory effect. For rs3740297, PASMC cell lines transfected with rs3740297 -C allele construct, miR-149 mimic, and antagomir miR-149 showed more sensitive change of the relative luciferase activity (Figure 3C, P < 0.001), which revealed its allelic binding effect to miR-149.

On the basis of results of the luciferase reporter assays, we further evaluated the regulatory role of rs3740297 on the expression of BMP9 in PASMC cell lines. As shown in Figure 5B, PASMC cell lines transfected with rs3740297 -C allele construct, miR-149 mimic, and antagomir miR-149 showed more sensitive change of the relative expression level of BMP9 (P < 0.001), while PASMC cell lines transfected with rs3740297 -T allele construct, miR-149 mimic, and antagomir miR-149, the relative expression level of BMP9 was not materially changed (P >0.05). Taking together, we can conclude that miR-149 could down-regulate the BMP9 expression with the existence of rs3740297 -C allele in PASMC cell line.

## DISCUSSION

In current study, we explored the associations between common variants of the *BMP9* gene and development of IPAH, using a case-control study among Chinese population. We found that minor alleles of *BMP9* rs3740297 and rs7923671 was significantly associated with increased plasma level of *BMP9*, and further decreased susceptibility of IPAH. Functional experiments by dual-luciferase reporter and RT-PCR assay also validated the findings above. To the best of our knowledge, this should be the first study which aims to explore the associations between common variants of the *BMP9* gene and development of IPAH.

The genetic basis of IPAH has been studied extensively, and the candidate gene approach of genetic association studies have identified many loci for the susceptibility of IPAH, although large part still remains unclear [13–18]. Different with heritable PAH which mainly caused by the mutation of the *BMPR2* gene, the genetic etiology of IPAH is more complex [19]. In 2009, as a landmark event, Yu et al. [20] revealed that -254 (C>G) SNP in the TRPC6 gene promoter may predispose individuals to an increased risk of IPAH by linking abnormal TRPC6 transcription to nuclear factor-kappaB. Following that, genetic variants in vascular endothelial growth factor (VEGF), Rho/Rac guanine nucleotide exchange factor 18 (ARHGEF18), lncRNA metastasis associated lung adenocarcinoma transcript 1 (MALAT1), superoxide dismutase 2 (SOD2), and many more were identified to be associated with the susceptibility of PAH consecutively [13, 14, 16, 17].

The *BMP9* gene, which was located at 10q11.22, owns two exons, and belongs to the transforming growth factor beta superfamily. It contains an N-terminal TGF-beta-like pro-peptide and a C-terminal transforming growth factor beta superfamily domain [21]. In Nikolic’s study, *BMP9* was identified as a sensitive and specific biomarker of PoPH, which could predict transplant-free survival and the presence of PAH in liver disease [10]. In current study, we also found the plasma level of *BMP9* in IPAH cases was significantly lower than that in controls, which indicated the potential as a plasma biomarker for IPAH. Besides, genetic variations of the *BMP9* gene have been linked to many diseases [9, 12, 22–24]. Ren et al. [22] found that allele C of *BMP9* rs7923671 (P = 0.0026; OR: 1.33, CI: 1.10–1.60) was significantly associated with risk of Ossification of the posterior longitudinal ligament (OPLL), which had the same direction with our findings.

In our study, *BMP9* rs3740297, which was predicted to locate at the binding site of miR-149, was significantly associated with increased plasma level of *BMP9* and decreased susceptibility of IPAH. We have 93.2% statistical power for rs3740297 to detect such a genetic association. Xie et al. [25] previously identified that miR-149 promotes human osteocarcinoma progression via targeting *BMP9*. In our study, we also tested whether interaction between miR-149 and *BMP9* existed in a rs3740297 allele-specific manner in PASMC cells, and confirmed that rs3740297 C allele (major allele) was a target of miR-149. Taking together, these results indicate that miR-149 is an inhibitor of *BMP9* [C] but not *BMP9* [T]. This means minor allele T of *BMP9* rs3740297 could significantly decrease susceptibility of IPAH in Chinese population, possibly by up-regulating *BMP9* expression through losing a miR-149 binding site.

## CONCLUSION

Conclusively, this study provides evidence for genetic associations between two specific variants in the *BMP9* gene and plasma level of *BMP9*, as well as the occurrence of IPAH. The results could contribute to the understanding of the mechanisms and molecular etiology of IPAH, which might lead to further biomedical or functional studies and recommendations for biomarkers of disease screening and personalized prevention that will finally reduce the incidence of this disease.

## MATERIALS AND METHODS

### Study population

We totally recruited 836 IPAH cases, and 900 frequency-matched healthy controls by age and gender. All patients were primarily diagnosed by pulmonary angiography and right heart catheterization. The diagnosis of IPAH was made by at least 2 experienced PAH experts according to WHO criteria that a mean pulmonary arterial pressure equal to or exceeding 25 mmHg associated with normal pulmonary capillary wedge pressure. Patients with heritable, and associated PAH were not included in the present study. Controls were enrolled from the normal physical examination during the same period. Each participant was interviewed face to face to collect the demographic information. After that, each subject was asked to donate 10 mL of peripheral venous blood using sodium ethylene diamine tetra -acetic acid (EDTA) tubes. After centrifugation at 3000 × g and 4 °C for 15 minutes, the supernatants and cell sediment were frozen at -80 °C, respectively. All patients provided informed consent for participation and recruitment protocols were approved by the institutional review boards of Zhongshan Hospital.

### TagSNP selection, DNA extraction and genotyping

The tagSNPs covering the *BMP9* gene and its 5kb flanking region were selected from 1000 genome CHB data (phase 3, minor allele frequency ≥ 5%, pairwise r2 ≥ 0.8) using the Haploview 4.2 software. Finally, five SNPs (rs3740297, rs7923671, rs73299055, rs3781226, rs4922508) were selected as the candidates. Genomic DNA was extracted from the stored cell sediment of the blood samples using a DNA Blood Mini Kit (Qiagen, Valencia, CA, USA) according to the manufacturer’s protocol. The genotyping for the SNPs was performed using the TaqMan allelic discrimination assay on an ABI 7900 system (Applied Biosystems Inc, Foster City, CA, USA). A random 10% samples for each of the five SNPs were repeatedly genotyped and the concordance rate was 100%.

### Enzyme-linked Immunosorbent Assay (ELISA) of *BMP9*

The plasma level of *BMP9* in 100 randomly selected IPAH cases and 100 randomly selected controls was measured using a Sandwich Enzyme-linked Immunosorbent assay (ELISA) kit (R&D Systems, Minneapolis, MN) according to the manufacturer’s protocol, respectively. The inter-assay coefficient of variation was 8%, and the intra-assay coefficient of variation was 5%. All the assays were replicated in three times independently.

### Luciferase reporter assay

Human pulmonary artery smooth muscle cell (PASMC) line was purchased from the Chinese Academy of Sciences Cell Bank (Shanghai, China), were cultured in Dulbecco’s Minimum Essential Medium (DMEM) medium. The plasmids were constructed by subcloning the wild types of rs3740297 and rs7923671 into the pGL-3 promoter vectors (Promega, Madison, WI, USA), respectively. Then, the mutant plasmids were created using the Quick Change Mutagenesis Kit (Stratagene, La Jolla, CA, USA). All constructs were confirmed by sequencing. All plasmids were transfected into PASMC cells using Lipofectamine 3000 reagent (Invitrogen). For rs3740297, mimic control, antagomir control, miR-149 mimic or antagomir miR-149 (GenePharma, Shanghai) were transfected in different groups, respectively. Finally, firefly and Renilla luciferase activity was measured on Synergy H1 microplate reader (BioTek Instruments, Winooski, VT, USA) using Dual-luciferase Reporter Assay System (Promega, Madison, WI, USA). Luciferase activity was normalized to Renilla activity to correct for differences in transfection efficiency. All the experiments were performed in triplicate.

### Real-Time polymerase chain reaction analyses of miR-149 and the BMP9 gene

Total RNA was extracted from plasma and PASMC cells using TRIzol reagent (Invitrogen, Carlsbad, CA, USA). Then cDNA was synthesized with M-MLV reverse transcriptase (Invitrogen). Real-time reverse transcription-polymerase chain reaction (RT-PCR) with SYBR Green assay (TaKaRa Biotechnology, Dalian, China) was performed to examine expression level of miR-149 and the BMP9 gene. Glyceraldehyde 3-phosphate dehydrogenase (GAPDH) was used as an internal control. The assay was conducted using the ABI 7300 system (Applied Biosystems). All reactions were done in triplicate and the expression level was calculated according to the equation 2^-Δ Δ Ct^.

### Statistical analysis

All analyses were conducted with SAS 9.3 software (SAS Institute, Inc., Cary, NC, USA), and a P value of < 0.05 for two-side was considered statistically significant. Differences in the distribution of selected demographic variables were evaluated by Pearson’s χ ^2^ test. For quantitative variables, statistical significance was determined using unpaired t-test or one-way ANOVA. Hardy-Weinberg equilibrium (HWE) for each SNP among controls was tested using a goodness of-fit χ ^2^ -test. The associations of each SNP with IPAH susceptibility were estimated by unconditional logistic regression analyses with odds ratios (ORs) and 95% confidence intervals (CIs), adjusted for age, gender, smoking and drinking status, and body mass index (BMI).
